# S100A8/S100A9 released by CD11b^+^Ly6G^+^ neutrophils activate MerTK^hi^Ly6c^lo^ reparative macrophages to delay denervated muscle atrophy

**DOI:** 10.1038/s12276-026-01813-0

**Published:** 2026-08-07

**Authors:** Yaoxian Xiang, Lei Zhu, Zhenwei Qiu, Zongqi You, Junxi Dai, Lei Xu, Junjian Jiang, Jianguang Xu

**Affiliations:** 1 Department of Hand Surgery, Huashan Hospital, Fudan University, Shanghai, People’s Republic of China; 2 NHC Key Laboratory of Hand Reconstruction (Fudan University), Shanghai, People’s Republic of China; 3 Shanghai Key Laboratory of Peripheral Nerve and Microsurgery, Shanghai, People’s Republic of China; 4 Shanghai Key Laboratory of Forensic Medicine, Shanghai Forensic Service Platform, Key Laboratory of Forensic Science, Ministry of Justice, Academy of Forensic Science, Shanghai, China; 5 Department of Neurosurgery, Shanghai Tenth People’s Hospital, Tongji University School of Medicine Shanghai, Shanghai, 200072 China

**Keywords:** Somatic system, Neutrophils, Monocytes and macrophages, Neuroimmunology, Experimental models of disease

## Abstract

Strategies to delay the process of denervated muscle atrophy are limited, in part because the microenvironmental function of the target muscle is not well understood. Immune microenvironment remodelling is one of the main changes of skeletal muscle environment after denervation. Our previous work found that denervated atrophic muscles recruit a large number of neutrophils and macrophages, and neutrophils can delay muscle atrophy, but the specific mechanism is still unclear. In this study, the expression of *S100a8/S100a9* was significantly up-regulated in the transcriptional group of early denervated skeletal muscle. Immunofluorescence, flow sorting and quantitative PCR analysis showed that S100A8/S100A9 mainly came from neutrophils. Further knockout of *S100a9* has the same effect as blocking neutrophils to aggravate muscle atrophy. Through MerTK blockers, it is clear that MerTK^hi^Ly6C^lo^ reparatory macrophages can delay the progression of muscle atrophy. After knockout of the *S100a9* gene, the mobilization of bone marrow haematopoietic stem cells decreased, the excretion of spleen monocytes was blocked, the number of peripheral blood monocytes decreased, the infiltration of monocytes/macrophages in target muscle decreased and the polarization of MerTK^hi^Ly6C^lo^ reparatory macrophages was blocked. It is clear that S100A9 regulates the polarization of MerTK^hi^Ly6C^lo^ reparative macrophages. Collectively, based on the interaction between neutrophils and macrophages, it is clear that S100A8/S100A9 participates in delaying the process of denervated muscle atrophy by promoting the polarization of MerTK^hi^Ly6C^lo^ reparatory macrophages, and provides a new immune target for the early delay and prevention of denervated muscle atrophy.

## S100A8 S100A9 Link Neutrophils to Muscle Atrophy Delay

Denervated skeletal-muscle atrophy poses a significant challenge in peripheral neurosurgery, with limited recovery of muscle function affecting patients’ quality of life. This study explores the role of the immune microenvironment, particularly neutrophils and macrophages, in delaying muscle atrophy. Researchers used male S100a9 knockout mice and C57BL/6 mice, performing sciatic-nerve transection to induce denervation. They found that neutrophils release S100A8/S100A9 proteins, which promote the polarization of reparative macrophages, marked by MerTK^hi^Ly6C^lo^, through the transcription factor Nr4a1. This polarization delays muscle atrophy by enhancing tissue repair. The study highlights the potential of targeting the S100A9/MerTK^+^ macrophage axis to develop new therapeutic strategies for muscle atrophy. Future research should focus on validating these findings in human tissues and exploring targeted delivery systems to enhance clinical applicability.

This summary was initially drafted using artificial intelligence, then revised and fact-checked by the author.

## Introduction

The effective treatment of denervated skeletal-muscle atrophy is still an unresolved challenge in peripheral neurosurgery. Long-term atrophy leads to almost no recovery of muscle function, which seriously affects the limb function and quality of life of patients. Therefore, exploring effective ways of promoting skeletal-muscle regeneration and repairing or delaying the process of skeletal muscle atrophy is urgent^1,2^. Skeletal muscle and its innervating peripheral nerves reside within a dynamic microenvironment. Alterations to this microenvironment, particularly within the immune compartment, may act as critical regulatory switches governing their functional interplay. By focusing on the microenvironment of injured peripheral nerves and atrophying skeletal-muscle cells, and by identifying key regulatory nodes that mediate their interactions, we can better map the real-world landscape of denervated muscle atrophy and develop effective strategies for counteracting this pathology. However, current research on the microenvironment of skeletal-muscle cells following denervation-induced atrophy remains scarce, with limited understanding of the role of the immune microenvironment in this process.

Neutrophils are key mediators of early tissue injury. They rapidly infiltrate sites of damage, where they can either exacerbate injury or facilitate repair. Both functions, however, depend on their capacity to directly interact with compromised tissues^3–6^. Although denervation does not cause direct damage or infection to muscle cells, and no local pathogens recruit neutrophils within denervated tissue, intravital spinning-disk confocal microscopy, in vivo imaging and 3D reconstruction revealed spatio-temporally specific neutrophil diffusion, instead of targeted recruitment toward denervated muscle.^7^

Previous studies have shown that whether after direct nerve or muscle injury, the inflammatory response mediated by immune cells is the main cause of secondary injury. Especially at the stage of acute inflammation, neutrophils aggravate tissue damage by secreting reactive oxygen species (ROS), proteolytic enzymes, inflammatory cytokines and chemokines^8^. However, the inhibition of key pro-inflammatory factors or the removal of innate immune cells significantly slows down the repair and functional recovery of peripheral-nerve injury^9^. Therefore, the function of innate immune cells seems to be much more complex than we previously thought, and immune cells have potential research value in the process of repairing nerve and muscle injury. This has a lot to do with the significant plasticity of immune cells and the phenotype of different functions^10,11^. At the same time, our previous studies have confirmed that neutrophils can delay the process of denervated muscle atrophy, but its potential molecular mechanism is still unclear^7^.

As stable heterodimer S100A8/S100A9, also known as calcitonin, S100A8 and S100A9 are stored in large quantities in neutrophils and released rapidly under inflammatory stimulation, acting as damage-related molecular patterns (DAMPs)^12^. Extracellular S100A8/S100A9 binds receptor for advanced glycation end products (RAGE)^13,14^ and Toll-like receptor 4 (TLR4)^15,16^, and acts as a powerful innate immune response activator in various diseases through immune and inflammatory components. Cytoplasmic phagocytic receptor-myeloid epithelial-reproductive receptor tyrosine kinase (MerTK) is mainly expressed in Ly6C^lo^ macrophages. It has been found that the phagocyte receptor MerTK is necessary for the repair of skeletal-muscle injury^17,18^. There are also studies that show that reparative macrophages in ischaemic myocardium were previously defined as CD11b^+^F4/80^+^ Ly6C^lo^ cells^19^. In three separate studies, lack of MerTK in myeloid cells led to repair defects^20–22^. Therefore, reparative macrophages can be defined as CD11b^+^F4/80^+^ Ly6C^lo^MerTK^hi^ cells. The study found that the number of reparative macrophages in the hearts of mice treated with S100A9 blockers decreased by about 50%^8^. The percentage of reparative Ly6C^lo^MerTK^hi^ in total CD11b^+^F4/80^+^ macrophages also decreased, suggesting that S100A9 blocked the phenotypic transition of mouse monocytes to reparative macrophages.

In view of the research background that S100A8/S100A9 can mediate the transformation of inflammatory macrophages to reparative macrophages and the basis of our completed work on neutrophils delaying atrophy^7^, this study determined that the recruitment of neutrophils in denervated skeletal muscle was the main source of S100A8/S100A9, and that S100A8/S100A9 played a role in delaying the process of denervated muscle atrophy by regulating the polarization of reparative macrophages. Starting from an analysis of spatio-temporally specific regulation of the immune microenvironment in denervated muscle atrophy, this study aims to elucidate the roles and key regulatory molecules underlying neutrophil-macrophage interactions in denervated muscle atrophy. Targeting these specific regulatory molecules may facilitate the development of novel preventive and therapeutic strategies to delay denervated skeletal muscle atrophy.

## Methods

### Experimental animals

Male *S100a9* knockout (KO) mice aged 6–8 weeks and weighing 22–25 g were purchased from the Model Organisms Center (Shanghai, China). Male C57BL/6 mice aged 6–8 weeks and weighing 22–25 g were purchased from Vital River Laboratory Animal Technology Co. (Zhejiang, China). Mice were housed in standard cages in a room at 23 °C and 50% relative humidity on a 12-h light:12-h dark cycle. Mice in both denervated groups were anaesthetized and subjected to unilateral sciatic nerve transection^23^. Briefly, after deep anaesthetization, a 0.5-cm-long portion of the sciatic nerve in the right hind leg of each mouse was resected; the two nerve ends were buried in the muscles, and the incision was closed using 4–0 absorbable sutures. The mice were randomly assigned to experimental groups for analysis at specific time points after denervation.

Mice were subjected to sciatic-nerve transection under isoflurane anaesthesia. Denervated muscles (gastrocnemius) and contralateral controls were harvested at 3 h, 6 h, 12 h, 1 day, 3 days and 7 days post-surgery (*n* = 4–6 per group). Tissue samples for RNA sequencing (6-h time point) were flash-frozen in liquid nitrogen, whereas samples for histology (at 3-h, 6-h, 12-h, 1-day, 3-day and 7-day time points) were fixed in 4% paraformaldehyde. Tissue samples for flow testing (3-day and 7-day time points) were placed in muscle-tissue culture medium.

Mice were randomly assigned to control, Den-7d+lgG or Den-7d+anti-MerTK groups (*n* = 6 per group). Mice were given 2.0 mg/kg intravenous polyclonal goat anti-MerTK antibody 1 h before denervation.

The animal study was reviewed and approved by the Institutional Animal Care and Use Committee of Huashan Hospital, Fudan University.

### Flow cytometry

For cell preparation and sorting, please refer to the supporting information. FVS dye (1 μl) was added to a suspension of single cells (1 ml; 3×10^6^ cells) slowly and evenly using a liquid transfer gun, and the suspension was then incubated at room temperature away from light for 30 min. Next, 3 ml of PBS was added to resuspend the cells, and the sample was centrifuged at 4 °C at 400 × g for 5 min, after which the supernatant was discarded. The cells were resuspended in PBS and then incubated with 5 μl of CD45-APC-Cy7, Ly6G-PE, CD11b-FITC, CD115-Pecpcy5, SCA-1-APC/AF647, c-Kit-PE, CD48-PE-CY7, CD150-BV605, Mer-BV421 and Ly6c-AF700 at room temperature in the absence of light. After incubation, 3 ml of PBS was added to resuspend the sample, which was centrifuged at 4 °C at 400 × *g* for 5 min, and the supernatant was discarded. Prior to detection, 200 μl of PBS was added to each tube. The data were analysed using FlowJo software.

### RNA sequencing

Total RNA was extracted from muscle-tissue samples using the miRNA Isolation Kit (mirVana; Thermo Fisher Scientific, Waltham, MA, USA; AM1561) according to the manufacturer’s protocol. The RNA integrity was evaluated using the Agilent 2100 Bioanalyzer (Agilent Technologies, Santa Clara, CA, USA), and samples with an RNA integrity number ≥ 6 were retained for analysis. Then, the libraries were constructed using the TruSeq Stranded messenger RNA (mRNA) LT Sample Prep Kit (Illumina, San Diego, CA, USA) according to the manufacturer’s instructions and sequenced on the Illumina HiSeq X Ten platform, generating 125/150-bp paired-end reads. Index-coded sample clustering was performed using the TruSeqPE Cluster Kit v3-cBot-HS (Illumina) on a cBot Cluster Generation System according to the manufacturer’s protocol. The Illumina HiSeq X platform was used to sequence the library preparations; 125-/150-bp paired-end reads and 50-bp single-end reads were generated. For other detailed methods, please refer to the supporting information. DEGs were identified using DESeq2 with thresholds of false discovery rate (FDR)-adjusted *P* < 0.05 and |log2(fold change)| ≥ 1. Raw *P* values were corrected for multiple testing using the Benjamini–Hochberg (FDR) method.

### Wet weight

At different time points after denervation, mice were anaesthetized, and the gastrocnemius muscles of both the left and right hind legs were removed, washed with saline and then weighed. The ratio of muscle weight loss was defined as the weight of the contralateral side minus the muscle weight of the nerve injury side divided by the weight of the contralateral side. The muscle samples were stored in 4% paraformaldehyde at −80 °C until use.

### Quantitative real-time PCR

The RNeasy kit (Qiagen, Valencia, CA, USA) was used to extract total RNA from the gastrocnemius muscle. The cDNA was synthesized by a first-strand cDNA synthesis kit with oligo dT primers (Invitrogen, Carlsbad, CA, USA) and was used for quantitative real-time PCR (MJ Research, Waltham, MA, USA). The thermal cycling conditions were as follows: 94 °C for 5 min; 35 cycles at 94 °C for 30 s, 55 °C for 45 s and 72 °C for 1 min; and 72 °C for 5 min. The relative expression level of the target gene was calculated by using the cycle threshold (Ct) value. The expression levels of *S100a8, S100a9, Trim63, Fbxo32* and *Nr4a1* were normalized to GAPDH levels. The primer sequences were as follows: *S100a8*: R, 5’- ATG CCA CAC CCA CTT TTA T-3’, F, 5’-ATG CCC TCT ACA AGA ATG ACT-3’; *S100a9*: R, 5’-CAT CTG AGA AGG TGC TTT GTT-3’, F, 5’-CTG GGC TTA CAC TGC TCT TAC-3’; *Trim63*: R, 5’-CCA TCA GGA ATC AGG CTA GA-3’, F, 5’-GTC AGG GGA CGA AGA CAA A-3’; *Fbxo32*: R, 5’-CTT CCC CCA AAG TAC AGT ATC-3’, F, 5’-GAG AAA GAA AGA CAT TCA GAA CA-3’; *Nr4a1*: R, 5’-ATC TCA ACC TCT TCC TTT CTG TA-3’, F, 5’-GTG ACC CCA CTA TTT GTC TTA TC-3’; *Mertk*: R, 5’-CTC CCG TTG AGA AAA GTT G-3’, F, 5’-CTG GAA GAT GTT GTG ATT GAC-3’; *GAPDH*: R, 5’-TGC CGT GAG TGG AGT CAT AC-3’, F, 5’-CCC GTA GAC AAA ATG GTG AA-3’.

### Western blot analysis

The frozen gastrocnemius muscle samples were homogenized in a radioimmunoprecipitation assay buffer containing 1 mM phenylmethylsulphonyl fluoride and the Protease Inhibitor Cocktail (Roche Applied Science). The lysates were centrifuged for 20 min at 12,000 × *g* (4 °C), and the protein level in the supernatant was quantified with a bicinchoninic acid assay kit (Beyotime). The proteins were separated by SDS–PAGE (Beyotime) and transferred to a polyvinylidene difluoride membrane (Beyotime) that was blocked with 5% non-fat dry milk in Tris-buffered saline at room temperature, followed by incubation with primary antibodies: mouse anti-MerTK (1:1000; Abcam, USA). After being washed three times, the membrane was incubated with the appropriate secondary antibody (Abcam) at room temperature for 1 h. The enhanced chemiluminescence detection reagent and an X-ray film were used to visualize the proteins.

### Immunohistochemistry

The expressions of the S100A8 and S100A9 in the gastrocnemius muscle were detected by immunohistochemistry. The sections were deparaffinized with xylene and rehydrated with ethanol, and antigen retrieval was performed in a 0.01 M citrate buffer (pH 6.0) in a pressure cooker, followed by natural cooling to room temperature. The sections were incubated in 0.3% hydrogen peroxide at room temperature for 10 min; goat serum was used to block the sections for 15 min at room temperature, and they were then incubated overnight at 4 °C with rabbit anti-S100A8 (1:100; Abcam, ab180735, UK) and rabbit anti-S100A9 (1:100; Abcam, ab92507, UK). This was followed by treatment with horseradish peroxidase-conjugated goat anti-rabbit IgG antibody (ABclonal, 33301ES60, Wuhan, China) for 30 min at room temperature. Immunodetection was performed by using a diaminobenzidine solution according to the manufacturer’s instructions. After being washed, the sections were counterstained, dehydrated and then coverslipped by using neutral gum sealant. Immunohistochemistry analysis was performed on 4 mice per group, with 3 tissue sections per mouse.

### Immunofluorescence

To identify the S100A8, S100A9, CD11b, Ly6G and F4/80 in the denervated muscles, double immunostaining was performed. The paraformaldehyde-fixed denervated muscles were incubated with the primary antibody followed by the secondary antibody and were subsequently mounted with DAPI. The primary antibodies included mouse anti-Ly6G (1:50; Santa, USA), rabbit anti-S100a8 (1:50; Abcam, ab180735, UK), rabbit anti-S100A9 (1:50; Abcam, ab92507, UK), rabbit anti-CD11b (1:200; Abcam, ab52632, UK) and rabbit anti-F4/80 (1:200; Santa, 28463-1-AP, USA). The number of positive cells was observed by fluorescence microscopy (1 × 71; Olympus, Japan) and quantified by Image J software. Tissues were harvested and processed for immunofluorescence imaging at 12 h post-treatment. Image acquisition parameters (e.g. exposure time, magnification) were standardized across all samples to ensure comparability.

### Haematoxylin–eosin staining

Gastrocnemius muscle samples from mice were fixed in 4% paraformaldehyde and embedded in paraffin. The samples were cut at a thickness of 5 µm, and the sections were stained with haematoxylin–eosin (Beyotime, Shanghai, China) to evaluate histopathological changes. The mean area, diameter and density of myofibres were determined by blinded analysis using Image-Pro Plus 6.0 software (National Institutes of Health, Bethesda, MD, USA) from six randomly captured images per mouse under each experimental condition. An EclipseCi-L camera microscope was used to select the target area of the tissue for ×400 imaging so that the tissue could fill the whole field of view as much as possible to ensure that the background light of each photo was consistent. After completion of imaging, Image-Pro Plus 6.0 analysis software was used to count the number of muscle fibres in three visual fields in each slice (mm²), to measure the diameter of five muscle fibres (mm), and to calculate the average muscle fibre area (mm²) (equal to the total area of muscle fibres/number of muscle fibres) and the muscle fibre density (equal to the number of muscle fibres/area of the visual field).

### Statistical analysis

Measurement variables are presented as the mean ± standard error of the mean (SEM), and categorical variables are presented as percentages. Differences between two groups were evaluated using the Chi-squared test. For comparisons among multiple groups, one-way analysis of variance (ANOVA) tests was conducted. Differences at different time points were evaluated by using the Friedman test. Statistical analyses were performed using SPSS software, v17.0 (SPSS Inc., Chicago, IL, USA). *P* values < 0.05 were considered statistically significant. Pathway enrichment analysis was performed and *P* values were adjusted for multiple testing via the Benjamini–Hochberg FDR method. Pathways with FDR-adjusted *P* < 0.05 were considered statistically significant.

## Results

### Denervation-challenged MerTK^hi^Ly6C^lo^ reparative macrophages delay the skeletal-muscle atrophy

**Fig. 1:**
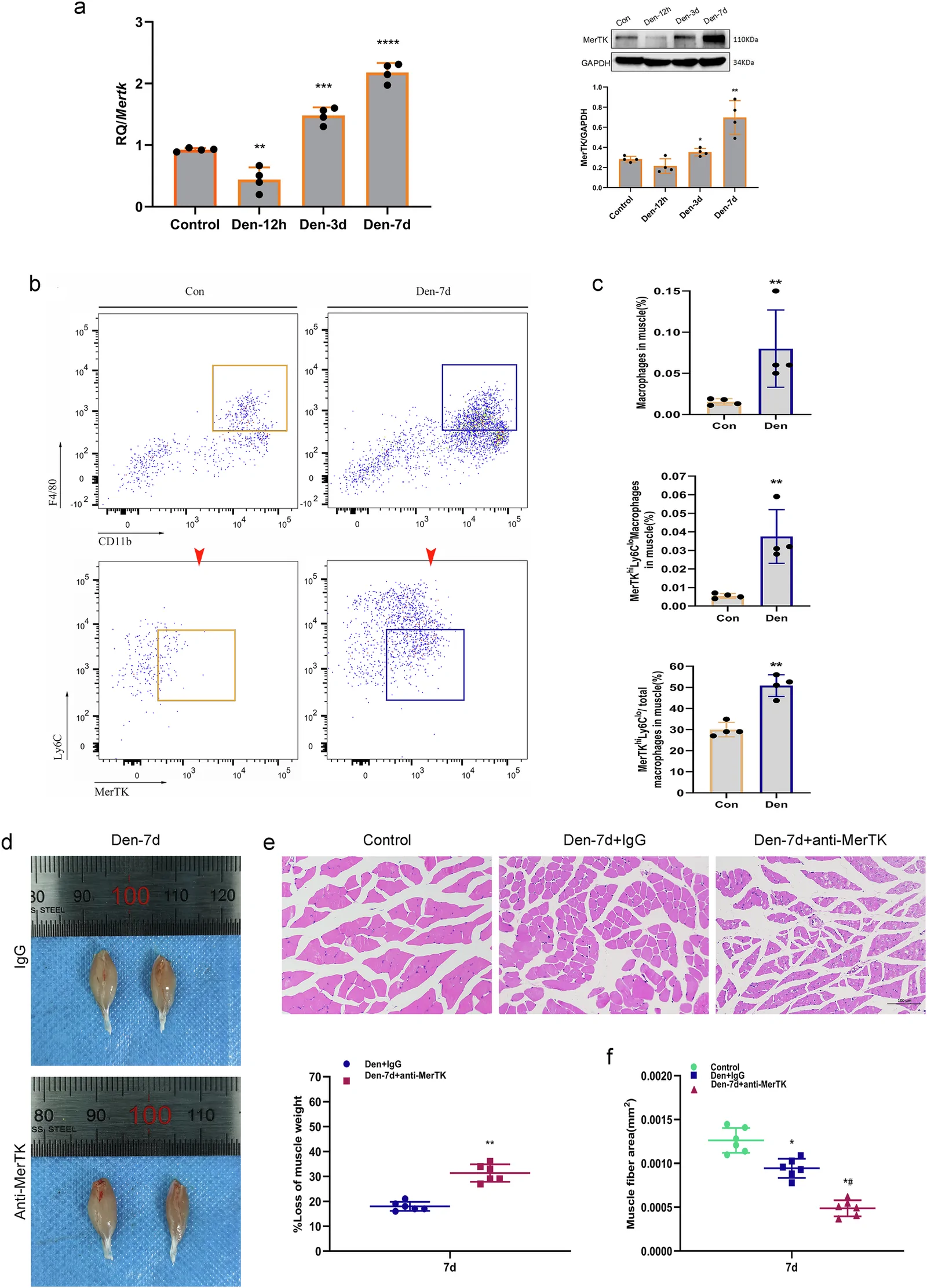
MerTK^hi^Ly6C^lo^ reparative macrophage increases in denervated muscle and delays muscle atrophy. **a** Elevated expression of the *Mertk* messenger RNA in the wild type (WT) group of mice after denervation was determined by quantitative PCR. The level of elevation of *Mertk* in mice in the Den-3d and Den-7d group was much higher than that of mice in the control group. **P < 0.01, ***P < 0.001, ****P < 0.0001 versus control (*n* = 4/group). MerTK protein levels determined by Western blotting showed a trend similar to that of the messenger RNA expression. *P < 0.05, **P < 0.01 versus control (*n* = 4/group). **b**, **c** Representative flow cytometry plots and counts of CD11b^+^F4/80^+^ and MerTK^hi^Ly6C^lo^ reparatory macrophages in control WT gastrocnemius muscle and muscle 7 d after denervation. **P < 0.01 versus Con (*n* = 4/group). Percentage of MerTK^hi^Ly6C^lo^ reparatory macrophages of total CD11b^+^F4/80^+^ macrophages in the skeletal muscle. **P < 0.01 versus Con (*n* = 4/group). **d** Representative images comparing muscle morphology of the denervated (right) versus contralateral control (left) gastrocnemius muscles treated with IgG or anti-MerTK, harvested at 7 days post-surgery. Appearance and loss of gastrocnemius muscle weight of WT control mice and anti-MerTK mice at 7 days post-denervation. **P < 0.01 versus Den (*n* = 6/group). **e**, **f** Haematoxylin–eosin staining of muscle tissue (×200) and mean ± SEM fibre area showing muscle atrophy that was ameliorated by MerTK^hi^Ly6C^lo^ reparatory macrophage. *P < 0.05 versus control; ^#^*P* < 0.05 versus Den (*n* = 6/group).

Compared with the control group, expressions of the MerTK protein as well as *Mertk* mRNA in the group of mice with muscle tissues after 3-day denervation were drastically higher (Fig. 1a). Next, we performed flow cytometry analyses of CD11b^+^F4/80^+^ macrophages and MerTK^hi^Ly6C^lo^ reparative macrophages in denervated gastrocnemius muscle. On the 7th day after denervation, the number of both of them that infiltrated into the target muscle had increased compared with the control group. Even the proportion of MerTK^hi^Ly6C^lo^ reparative macrophages in CD11b^+^F4/80^+^ macrophages had increased (Fig. 1b, c). To explore the function of MerTK^hi^Ly6C^lo^ reparative macrophages in the denervated model, mice were given 2.0 mg/kg intravenous polyclonal goat anti-mouse Mer antibody or control goat IgG antibody 1 h before denervation. Denervation-induced loss of muscle weight was significantly aggravated 7 days after injection of anti-mouse Mer anti-body (Fig. 1d, e). In addition, the mean fibre area of the gastrocnemius muscle was smaller than that of control goat IgG antibody-injected mice 7 days post-denervation (Fig. 1f).

These results suggest that MerTK^hi^Ly6C^lo^ reparative macrophages delay atrophy of denervated muscle in the first 7 days.

### Denervation increases expression of *S100a8/S100a9* in the denervated skeletal muscle

**Fig. 2:**
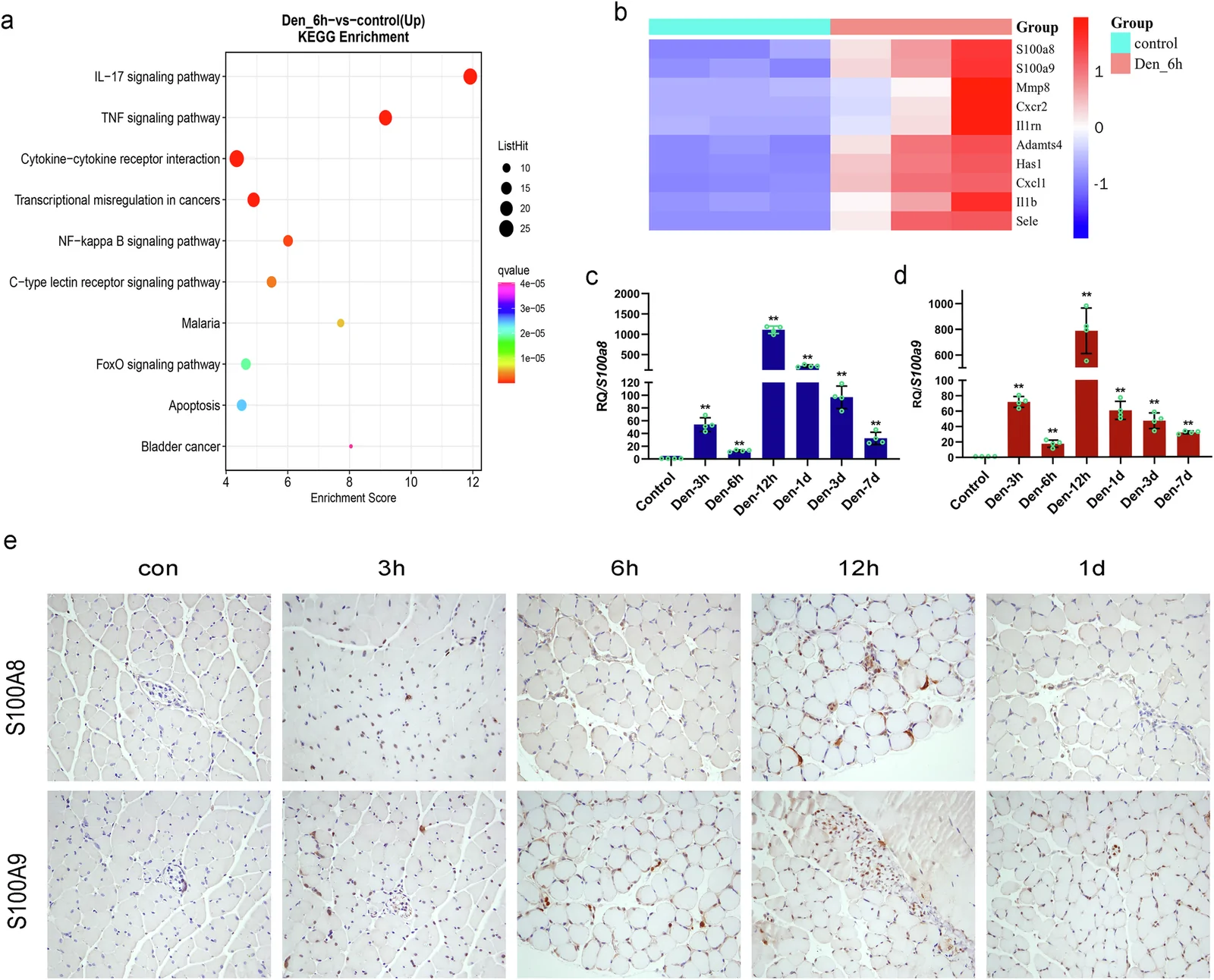
Denervation induces *S100a8/S100a9* expression in denervated skeletal muscle. **a** Top 10 Kyoto Encyclopedia of Genes and Genomes pathways of differentially expressed genes (DEGs). **b** Heatmap of the top 10 upregulated DEGs in gastrocnemius muscle from control wild type mice and 6 h after denervation. Heatmap colours indicate directionality (red: increased; blue: decreased). DEGs were defined as genes with false discovery rate-adjusted *P* < 0.05 and |log2(fold change)| ≥ 1. **c**, **d** *S100a8****/****S100a9* messenger RNA expression in control wild type mouse gastrocnemius muscle and after denervation. **P < 0.05 vs control (*n* = 4/group). **e** Compared with muscles of mice in the control group, the positive expression of S100A8/S100A9 increased in the denervated muscles of the Den-3h, Den-6h, Den-12h, Den-1d groups of mice.

To investigate the molecular mechanism of early pathological changes of denervated muscle atrophy, the gastrocnemius muscle transcriptome data for 6 h post-denervation were mined, which revealed that: 1) the top 10 enriched Kyoto Encyclopedia of Genes and Genomes pathways of the DEGs included the “IL-17 signalling pathway” (Fig. 2a); and 2) *S100a8* and *S100a9* were among the top 10 upregulated genes (Fig. 2b). Quantitative reverse-transcription polymerase chain reaction assays showed that muscle *S100a8* and *S100a9* expression increased significantly 3 h post-denervation, with peak *S100a8* and *S100a9* expression at 12 h (Fig. 2c, d). The results of immunohistochemistry confirmed this change (Fig. 2e). Collectively, these data suggest that the expression of *S100a8/S100a9* increases in the early stage of denervated skeletal muscle. Intriguingly, their change level was consistent with the change trend of the number of neutrophils in this model in our previous study^7^.

### S100A8/S100A9 was expressed primarily in CD11b^+^Ly6G^+^ neutrophils infiltrating the denervated muscle

**Fig. 3:**
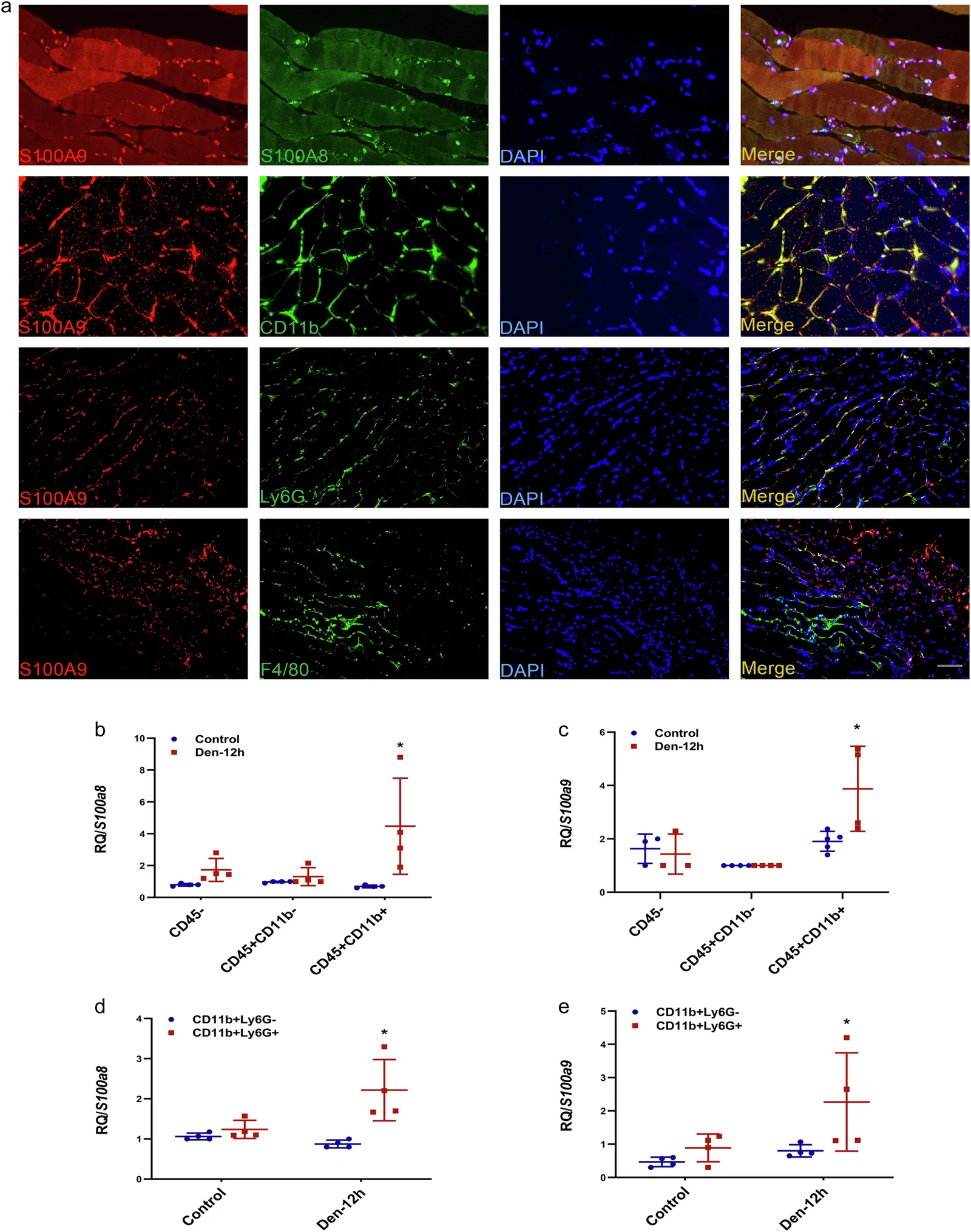
*S100a8/S100a9* was expressed primarily in CD11b^+^Ly6G^+^ neutrophils infiltrating the denervated muscle. **a** Representative immunofluorescence images in skeletal muscle at 12 h post-treatment. Immunofluorescence staining showed S100A9 (red), S100A8 (CD11b), Ly6G and F4/80 (green) in gastrocnemius muscle. DAPI staining showed that the nucleus was blue (×200). **b**, **c** Level of S100a8/a9 was measured by real-time polymerase chain reaction in CD45^−^ cells, CD45^+^CD11b^−^ cells and CD45^+^CD11b^+^ cells. In controls and 12 h after denervation, the infiltrated neutrophils were isolated by magnetic beads. *P < 0.05 vs control. **d**, **e** Levels of *S100a8/S100a9* messenger RNA levels in CD45^+^CD11b^+^Ly6g^+^ cells and CD45^+^CD11b^+^Ly6G^−^ cells sorted from peripheral blood leukocytes. *P < 0.05 vs control.

S100A8/S100A9 was expressed in myeloid cells such as neutrophils and monocytes. In order to determine which cell types expressed S100A8/S100A9 in denervated gastrocnemius muscle, double immunofluorescence staining showed that S100A8 was co-located with S100A9, CD11b and Ly6G, respectively. However, S100A9 was less co-located with F4/80^+^ macrophages (Fig. 3a). Magnetic beads were used to sort the denervated gastrocnemius CD45^−^ cells, CD45^+^CD11b^−^ cells and CD45^+^CD11b^+^ cells after 12 h of denervation. The expression of S100a8/a9 mRNA was highest in CD45^+^CD11b^+^ cells but was unchanged in CD45^−^ and CD11b^−^ cells (Fig. 3b, c). The CD45^+^CD11b^+^Ly6G^+^ cells and CD45^+^CD11b^+^Ly6G^−^ cells were isolated from peripheral blood leukocytes after 12 h of denervation. The S100a8/a9 mRNA expression of CD45^+^CD11b^+^Ly6G^+^ cells was higher than that of CD45^+^CD11b^+^Ly6G^−^ cells (Fig. 3d, e). Therefore, S100A8/S100A9 mainly comes from neutrophils in denervated gastrocnemius muscle.

### The genetic *S100a9* deficiency contributes to denervated muscle atrophy

**Fig. 4:**
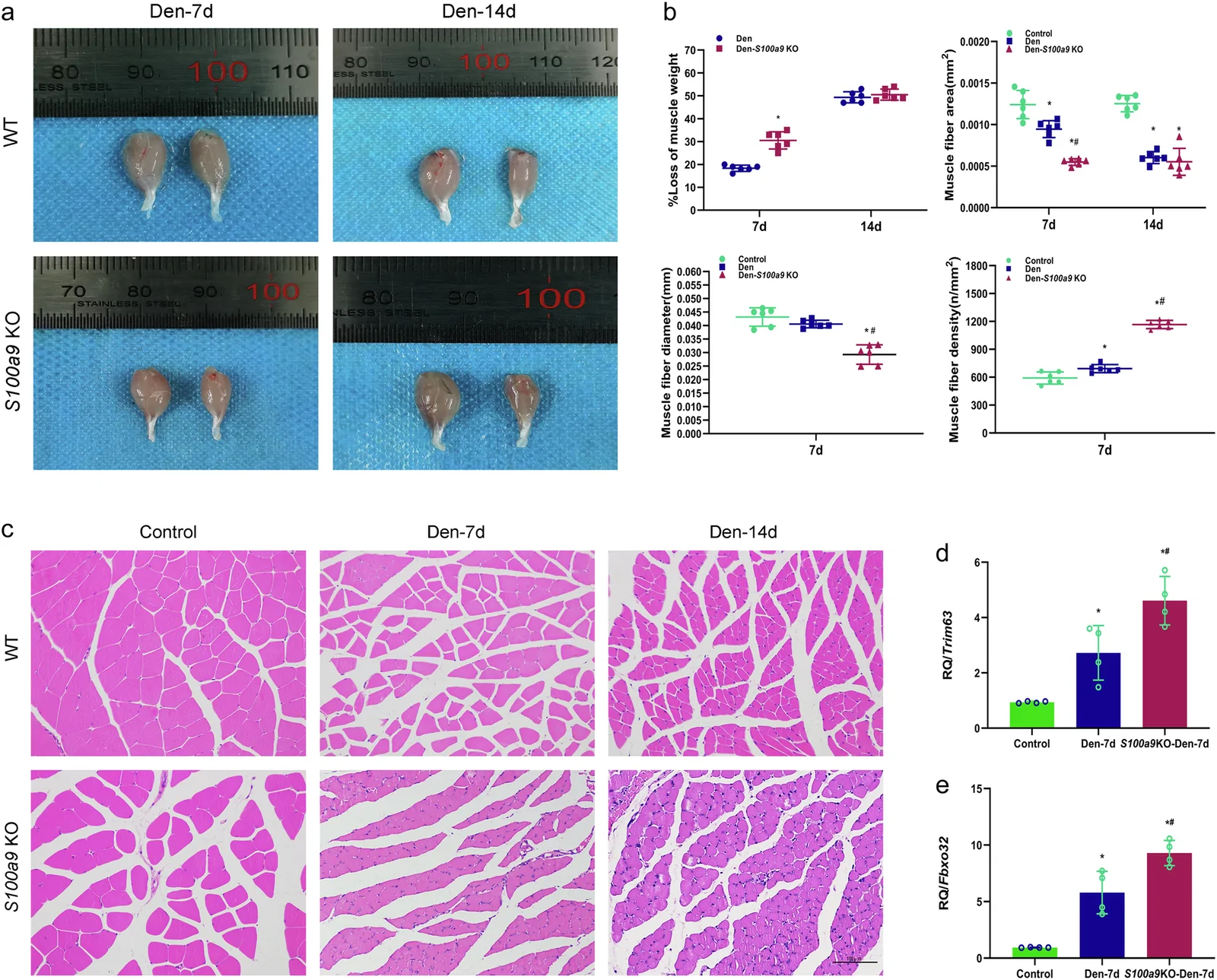
Aggravate denervated muscle atrophy using *S100a9* knockout. **a–c** Representative images comparing muscle morphology of the denervated (right) versus contralateral control (left) gastrocnemius muscles from wild type (WT) and *S100a9* knockout (KO) mice, analysed at 7 and 14 days post-surgery. Appearance and loss of gastrocnemius muscle weight of WT control mice, the *S100a9* KO and WT mice at 7 and 14 days post-denervation. *P < 0.05 versus Den (*n* = 6/group). Haematoxylin–eosin staining of muscle tissue (×200), mean ± SEM fibre area, mean fibre diameter and mean fibre density showing muscle atrophy that was aggravated by *S100a9* KO. *P < 0.05 versus control; ^#^*P* < 0.05 versus Den (*n* = 6/group). **d**, **e** The expressio*n* of *Trim63* and *Fbxo32* messenger RNA in the *S100a9* KO and WT mice at 7 days post-denervation was determined by quantitative PCR, showing that muscle atrophy was aggravated by *S100a9* KO.

To gain insights into the function of elevated *S100a9* in denervated muscle atrophy, the loss-of-function approach was used in the model of denervated mice. The targeted deletion of *S100a9* in the *S100a9* KO mice resulted in a loss in *S100a9*. After 7 and 14 days of inducing sciatic injury in the *S100a9* KO mouse group, the denervation-induced loss of muscle weight was significantly aggravated after 7 days, the mean fibre area and the mean fibro-diameter of the gastrocnemius muscles were lower, and their mean fibre density was larger than in the WT mice after 7 days, but there was no change within 14 days after 7 days (Fig. 4a–c4). Levels of atrophy-related markers such as *Trim63* and *Fbxo32* in mice with denervated muscle tissues increased significantly compared with the control group. The *Trim63* and *Fbxo32* levels of the *S100a9* KO-Den-7d mouse group were much higher than those of the Den-7d mouse group (Figs. 4d, e). Our results suggest that *S100a9* deficiency can aggravate denervated muscle atrophy.

### Haematopoiesis and phagocyte egression from the myeloid compartments into the blood are blunted by *S100a9* knockout

**Fig. 5:**
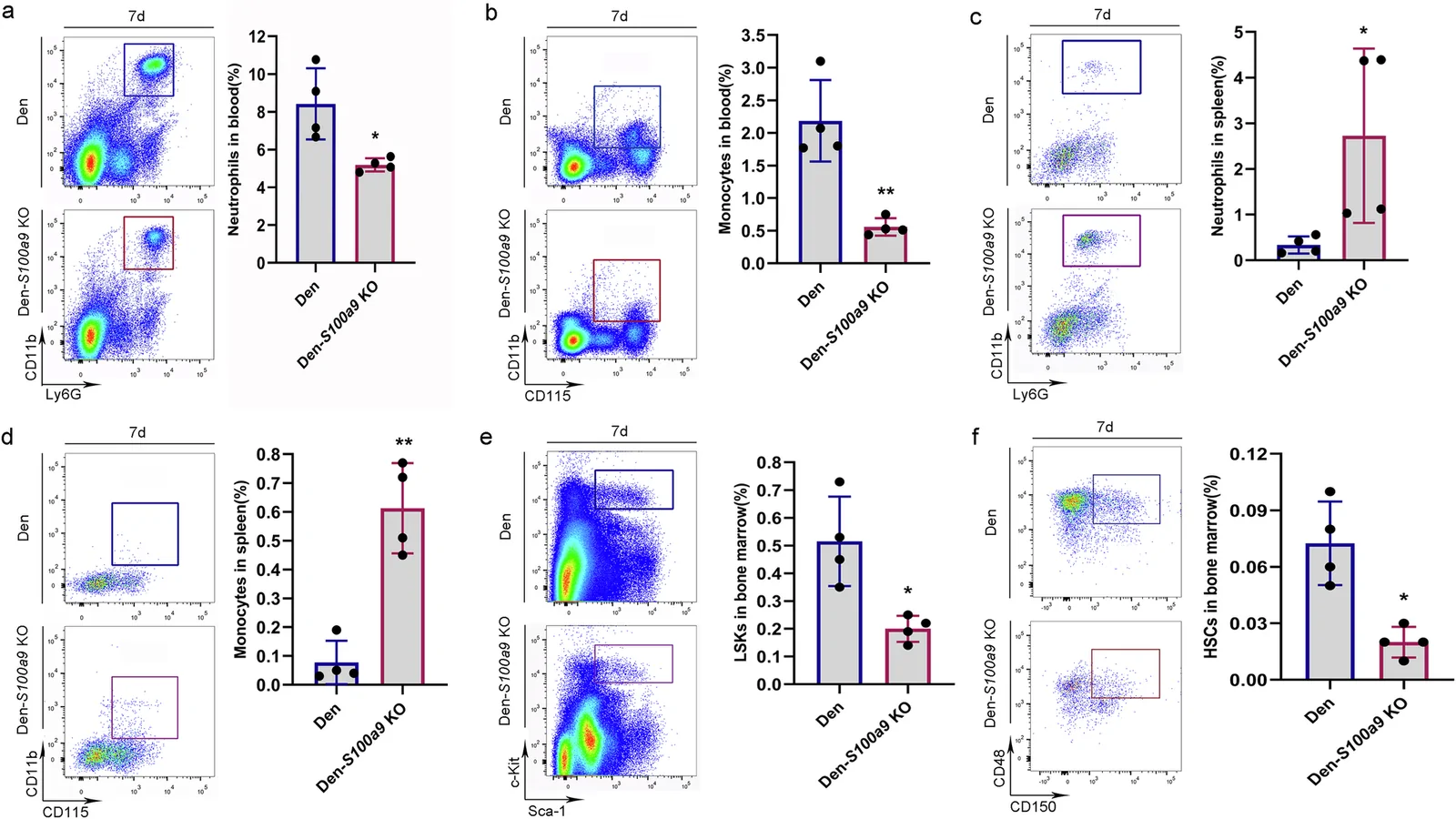
*S100a9* deficiency inhibits myeloid cell production and trafficking. Representative flow cytometry plots and counts of CD11b^+^Ly6G^+^ neutrophils and CD11b^+^CD115^+^ monocytes in blood (**a, b**) and spleen (**c, d**) of mice treated with PBS (Den) or the *S100a9* deficiency (Den-*S100a9* KO) for 7 days after denervation. **e**, **f** Representative flow cytometry plots and counts of Lin^−^ Sca^+^ Kit^+^ (LSK) cell and Lin^−^Sca^+^Kit^+^CD150^+^CD48^−^ haematopoietic stem cells (HSCs) in the bone marrow of mice treated with PBS (Den) or the *S100a9* deficiency (Den-*S100a9* KO) for 7 days after denervation. *P < 0.05, **P < 0.01 versus Den (*n* = 4/group).

We tried to verify the effect of *S100a9* on the effect of *S100a9* KO on bone-marrow cell response and the polarization of MerTK^hi^Ly6c^lo^ reparative macrophages. We measured the count of myeloid cells in blood, spleen and bone marrow of the *S100a9* KO group on the 7th day after denervation and compared it with the WT control group of the denervated model. In blood, the number of neutrophils and monocytes decreased significantly after *S100a9* KO (Fig. 5a, b). Meanwhile, *S100a9* KO causes neutrophils and monocytes to gather in the spleen and block peripheral excretion (Fig. 5c, d). In addition, *S100a9* KO inhibits the proliferation of haematopoietic stem cells and progenitor cells and haematopoietic stem cells in bone marrow (Fig. 5e, f). The source of conversion precursors of MerTK^hi^Ly6c^lo^ reparative macrophages was reduced and the polarization was blocked. Therefore, the mobilization source, transit station and endpoints of immune cells and precursors from the bone marrow to the periphery blood and target tissue are blocked.

### *S100a9* is involved in MerTK^hi^Ly6C^lo^ macrophage polarization in the microenvironment of denervated muscle cells

**Fig. 6:**
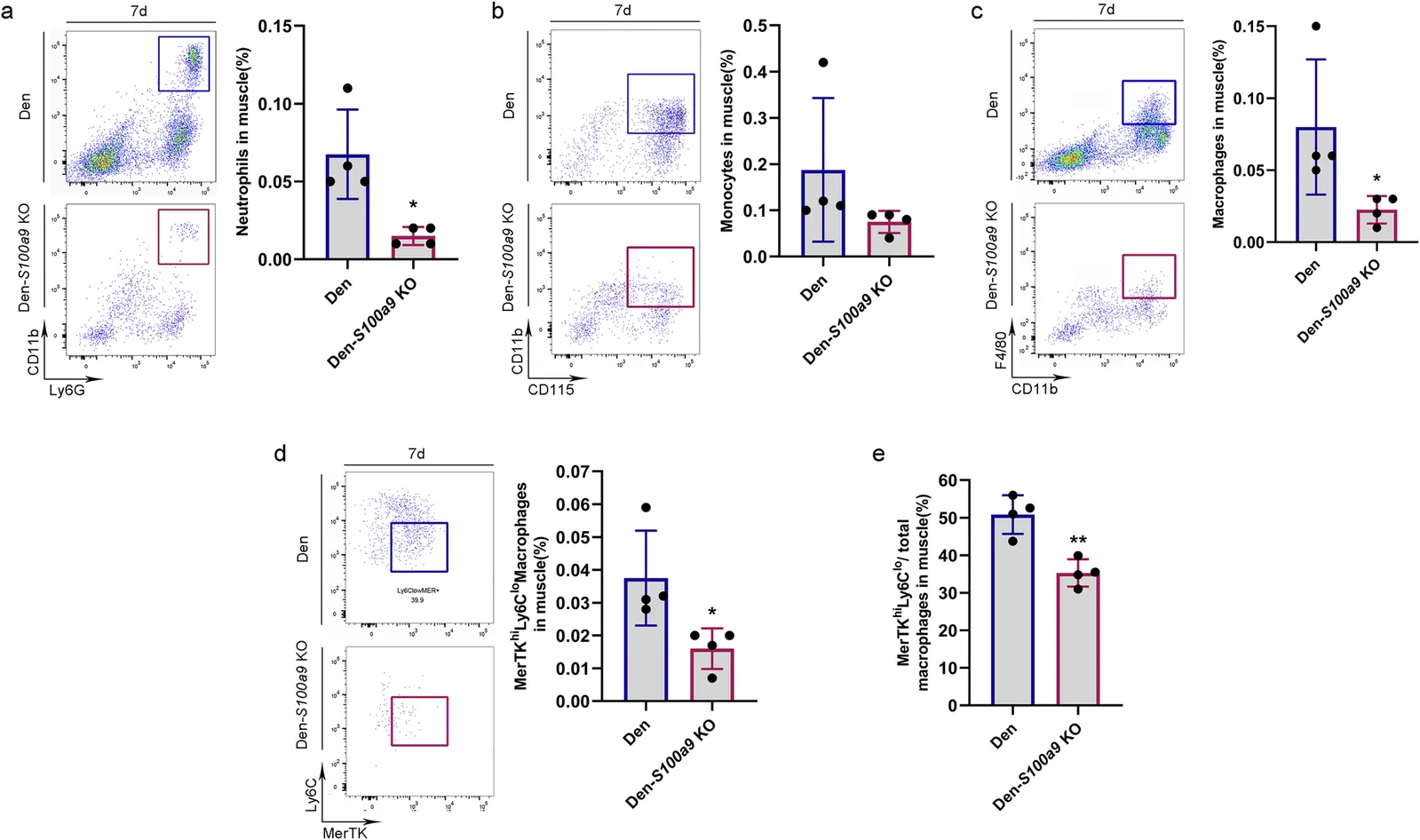
*S100a9* deficiency reduces the numbers of myeloid cells in denervated muscle. Representative flow cytometry plots and counts of CD11b^+^Ly6G^+^ neutrophils (**a**) and CD11b^+^CD115^+^ monocytes (**b**) CD11b^+^F4/80^+^ total macrophages (**c**) and CD11b^+^F4/80^+^Ly6c^lo^ reparatory macrophages expressing the efferocytosis receptor MerTK (**d**) in the denervated muscle of mice treated with PBS (Den) or the *S100a9* deficiency (Den-*S100a9* KO) for 7 days after denervation. **e** Percentage of MerTK^hi^Ly6C^lo^ reparatory macrophages of total CD11b^+^F4/80^+^ macrophages in the skeletal muscle. *P < 0.05, **P < 0.01 versus Den (*n* = 4/group).

Next, we studied the effect of *S100a9* KO on the existence of myeloid cells in denervated atrophic skeletal muscle. On the 7th day after denervation, the number of neutrophils infiltrated into the target muscle decreased in the *S100a9* KO group (Fig. 6a). *S100a9* KO also significantly reduced the presence of monocytes in target muscle (Fig. 6b). The total number of CD11b^+^F4/80^+^ macrophages in target muscle also decreased significantly (Fig. 6c). We define the reparative macrophages in denervated skeletal muscle as CD11b^+^F4/80^+^MerTK^hi^Ly6C^lo^ cells. We found that the number of reparative macrophages in the target muscle of *S100a9* KO mice decreased by about 50% compared with the control group (Fig. 6d). The percentage of MerTK^hi^Ly6C^lo^ reparative macrophages in total CD11b^+^F4/80^+^ macrophages also decreased, suggesting that the phenotypic transition of monocytes to reparative macrophages in *S100a9* KO-treated mice was impaired (Fig. 6e).

### *S100a9* activates reparative macrophages through upregulating *Nr4a1* between 3 days and 7 days

**Fig. 7:**
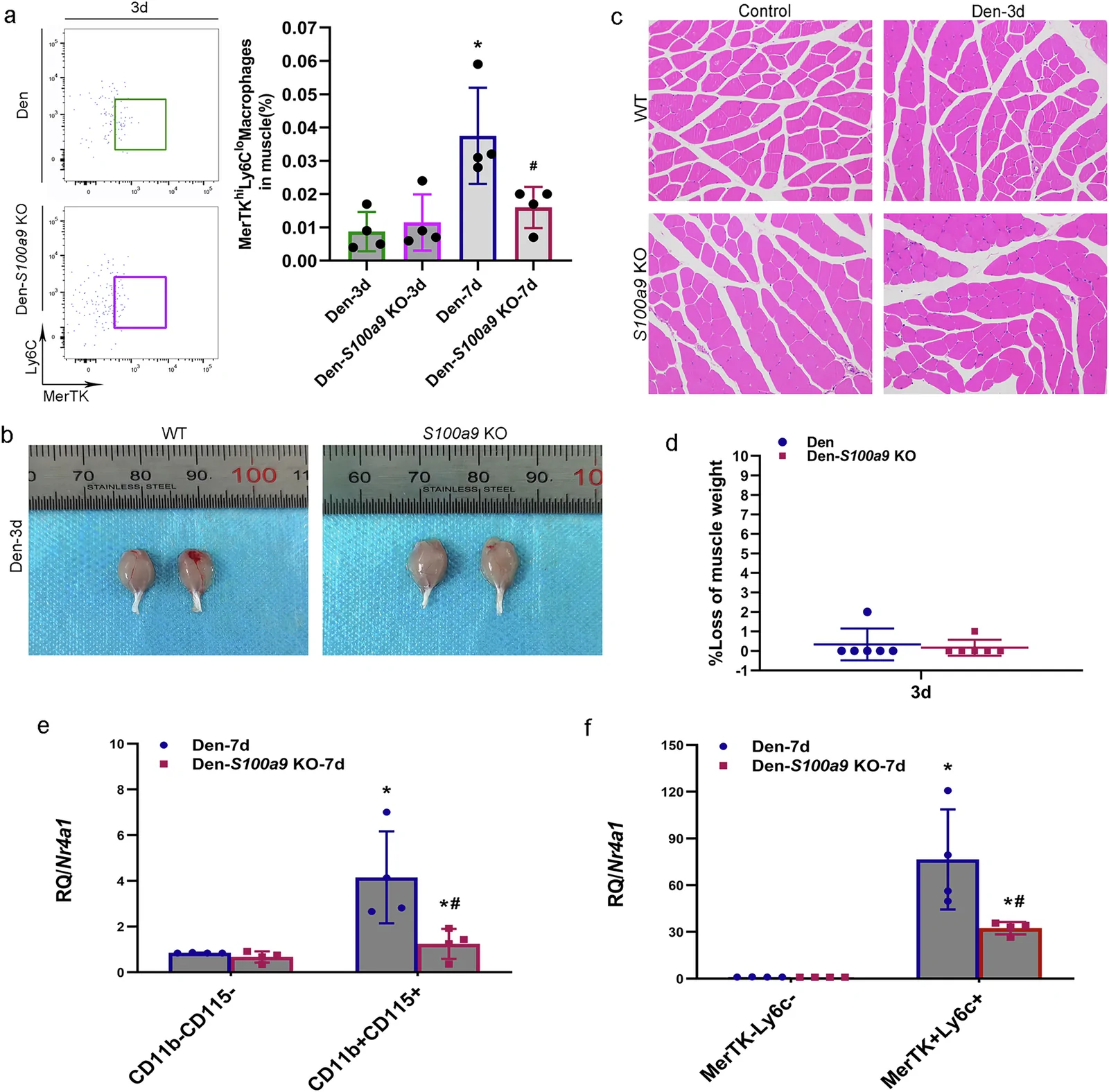
*S100a9* activates reparative macrophages through upregulating *Nr4a1* between 3 days and 7 days. **a** CD11b^+^F4/80^+^Ly6c^lo^ reparative macrophages expressing the efferocytosis receptor MerTK in the denervated muscle of mice treated with PBS (Den) or the *S100a9* deficiency (Den-*S100a9* knockout, KO) for 3 or 7 days after denervation. **b–d** Representative images comparing muscle morphology of the denervated (right) versus contralateral control (left) gastrocnemius muscles, harvested at 3 days post-surgery. Appearance, haematoxylin–eosin staining of muscle tissue (×200) and loss of gastrocnemius muscle weight of wild type (WT) control mice, the *S100a9* KO and WT mice at 3 days post-denervation. **e** Level of *Nr4a1* was measured by real-time PCR in CD11b^−^CD115^−^ cells and CD11b^+^CD115^+^ cells. In the Den-7d and Den-*S100a9* KO-7d blood groups, the infiltrated monocytes were isolated by magnetic beads. *P < 0.05 vs Den-7d or Den-*S100a9* KO-7d groups in CD11b^−^CD115^−^ cells, ^#^*P* < 0.05 vs Den-7d groups in CD11b^+^CD115^+^ cells. **f** Levels of *Nr4a1* messenger RNA levels in MerTK^−^Ly6c^−^ cells and MerTK^+^Ly6c^+^ cells sorted from denervated muscle. *P < 0.05 vs Den-7d or Den-*S100a9* KO-7d groups in MerTK^−^Ly6c^−^ cells, ^#^*P* < 0.05 vs Den-7d groups in MerTK^+^Ly6c^+^ cells.

Under homeostatic conditions, the steady-state or non-classical Ly6c^lo^ monocytes are derived from classical or inflammatory Ly6c^hi^ monocytes mediated by transcription factor *Nr4a1* (ref.^24^). In addition, *Nr4a1* plays an important role in the differentiation of Ly6c^hi^ monocytes into reparative macrophages. In our study, flow cytometry was used to detect MerTK^hi^Ly6C^lo^ reparative macrophages in WT and *S100a9* KO model muscle after 3 days and 7 days of denervation, respectively. It was found that the number of MerTK^hi^Ly6C^lo^ reparative macrophages in WT and *S100a9* KO denervated 3-day groups was very small, and that there was no difference between the two groups, whereas the number of MerTK^hi^Ly6C^lo^ reparative macrophages in the *S100a9* KO denervated 7-day group was significantly lower than that in the WT denervated 7-day group, indicating that MerTK^hi^Ly6C^lo^ reparative macrophages began to increase gradually in the target muscle after 3 days of denervation (Fig. 7a). Therefore, the appearance, the denervation-induced loss of muscle weight and haematoxylin–eosin staining of the target muscle of WT and *S100a9* KO model muscle after 3 days of denervation were evaluated. The results showed that *S100a9* KO had no effect on delaying the progress of denervated muscle atrophy within 3 days (Fig. 7b–d), which was related to no increase in reparative macrophages within 3 days. In order to study the effect of *S100a9* KO on the expression of *Nr4a1* in vivo, we sorted MerTK^hi^Ly6C^lo^ macrophages and other cells in gastrocnemius muscle 7 days after denervation by immunomagnetic beads and detected the level of *Nr4a1* by PCR. The expression of *Nr4a1* in blood monocytes of *S100a9* KO-treated mice decreased significantly (Fig. 7e), and there was little expression in WT and *S100a9* KO model groups of other cells. The Nr4a1 level was also significantly lower in gastrocnemius MerTKhiLy6Clo reparative macrophages of S100a9 KO-treated mice; only weak Nr4a1 signals were observed in other cell subsets isolated from WT and S100a9 KO model groups. (Fig. 7f).

## Discussion

**Fig. 8:**
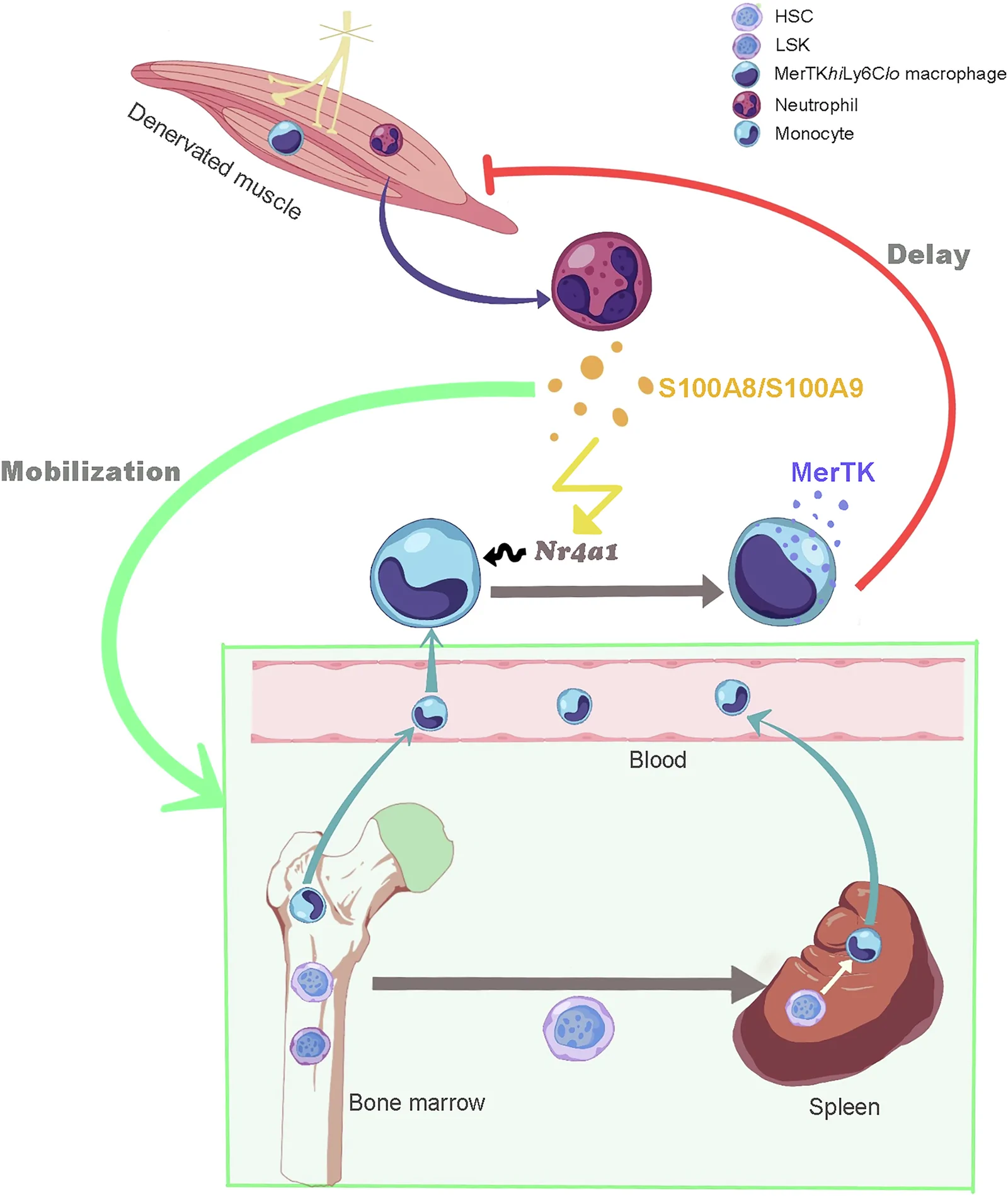
Following denervation-induced muscle atrophy, a disruption of immune homeostasis triggers remodelling of the local immune microenvironment. Neutrophils are recruited to the denervated muscle, where they release S100A8/S100A9 to drive the polarization of MerTK^hi^Ly6C^lo^ reparative macrophages through *Nr4a1*. S100A9-mediated Mertk activation in macrophages is a key step in mitigating denervation-induced muscle atrophy. This macrophage subset orchestrates tissue-repair mechanisms that contribute to the attenuation of denervation-associated muscle atrophy.

Our study found for the first time that the immune balance was broken, the immune microenvironment was remodelled and neutrophils were transported and recruited into muscle to release S100A8/S100A9 after denervation. Systemically, S100A9 stimulates the production of myeloid cells and the transport from bone marrow to denervated target muscles. Locally, S100A9 promotes the polarization of MerTK^hi^Ly6C^lo^ reparative macrophages and delays the process of denervated muscle atrophy by up-regulating the level and activity of transcription factor *Nr4a1*. S100A8/S100A9, which has become a key bridge between neutrophils and macrophages in target muscle, is one of the most important response factors to delay the process of denervated muscle atrophy (Fig. 8).

Our previous study found that muscle atrophy was aggravated within 1 week after administration of neutrophil blockers in denervated model mice^7^. In order to further explore the molecular mechanism of neutrophil delaying muscle atrophy, we analysed the skeletal muscle after 6 h of denervation by transcriptome sequencing. The Kyoto Encyclopedia of Genes and Genomes signal pathway analysis (Top 10) showed that the classical IL-17 signal pathway of neutrophils was significantly enriched, and then differential gene analysis showed that the expression of target genes *S100a8* and *S100a9* in this signal pathway was significantly up-regulated. Neutrophils are the most important source of extracellular *S100a8/S100a9* (refs.^25,26^). We found that the expression of S100a8/a9 increased significantly after denervation, and its change was consistent with the change in neutrophil recruitment^7^. But these proteins can also be secreted by other types of cells such as monocytes, macrophages, endothelial cells and platelets^27^. We further confirmed by immunofluorescence co-localization and flow sorting that S100A8/S100A9 in denervated muscle mainly came from recruited neutrophils. Knockout of *S100a9* gene had the same effect as blocking neutrophils to aggravate muscle atrophy, and it only played a role within 1 week. Therefore, we focused on the functional regulation of protein S100A8/S100A9.

Macrophages play a key role in skeletal-muscle healing/regeneration^28^. Monocytes/macrophages constitute the primary inflammatory cells recruited to acutely damaged skeletal muscle. In the early phase, pro-inflammatory macrophages with phagocytic activity predominate; these cells subsequently differentiate into reparative macrophages that promote muscle regeneration and myofibre restoration.^29^. Therefore, the transformation of macrophage subsets is essential for muscle regeneration and repair^30^. In our previous single-cell sequencing analysis of denervated human skeletal-muscle samples, we found that there was mainly recruitment of a large number of neutrophils and macrophages. After denervation, the interaction between neutrophils and macrophages recruited from the target muscle, especially with reparative macrophages, is particularly important for explaining the process of muscle atrophy. Previous studies on myocardial injury have found that neutrophils take the lead in promoting the recruitment of inflammatory macrophages^31^, and then up-regulate transcription factor *Nr4a1* to transform inflammatory macrophages into reparative macrophages to repair injured cardiomyocytes.

Therefore, the role of neutrophils at different stages of denervation is complex, and a detailed understanding of the pathogenesis and repair mechanism triggered by neutrophil mediators is a prerequisite for the development of neutrophil-targeted therapy. In addition, in the latest myocardial injury S100A9 blocking experiment, it was found that *Nr4a1* plays an important role in the differentiation of Ly6c^hi^ monocytes into reparative macrophages after myocardial ischaemia. At the same time, *Nr4a1* initiated the transplant of transgenic bone marrow expressing fluorescent EGFP protein into C57BL/6 mice to induce myocardial infarction. Flow cytometry showed that the expression of *Nr4a1* in blood Ly6c^hi^ monocytes of mice receiving S100A9 blockers was significantly decreased. Therefore, S100A9 promotes the transformation of inflammatory monocytes into reparative macrophages by activating *Nr4a1*. Previous studies have shown that phagocyte receptor MerTK plays an indispensable role in the repair of skeletal-muscle injury^17^. MerTK is mainly expressed in Ly6C^lo^ macrophages and participates in the regulation of macrophage phagocytosis. Its binding with ligands can participate in the phosphatidylserine connection of the “Eat-me” signal on the target apoptotic cells^32^. *Mertk* mutant mice could not effectively eliminate apoptotic cells. Phagocytosis in vitro showed that this defect was caused by macrophages^33^. Therefore, MerTK plays a major role in regulating macrophage clearance of apoptotic cells to repair tissue^34^.

In this study, we blocked the expression of MerTK by blockers, and found that MerTK^+^ repair macrophages could delay the progress of muscle atrophy and play a repair role in the denervated model. We further established a denervated model by *S100a9* KO mice. Through flow analysis of bone marrow, spleen, peripheral blood and denervated gastrocnemius muscle, we found that bone-marrow haematopoietic stem-cell mobilization decreased, spleen neutrophils and monocytes were blocked, peripheral blood neutrophils and monocytes decreased, and muscle neutrophils and monocytes/macrophages recruitment decreased. The source of conversion precursors of MerTK^hi^Ly6C^lo^ reparative macrophages decreased and the polarization was blocked. This would clarify whether macrophage recruitment is dependent on S100A8/S100A9. We want to interpret our findings cautiously, emphasizing the correlative nature of the data and citing prior studies that link S100A8/S100A9 to macrophage recruitment^8^. In order to reveal *S100a9* as a stimulator of *Nr4a1* expression and activity, we further knocked out the *S100a9* gene, isolated monocytes from the peripheral blood flow of denervated mice and isolated MerTK^+^ reparative macrophages from denervated gastrocnemius muscle. The expression of *Nr4a1* in these cells decreased significantly.

From the point of view of treatment, in the tissue directly injured, the level of *S100a8/S100a9* increased significantly within 12 h, and played the role of damage molecule in the target tissue^35,36^. In our model, the level of *S100a8/S100a9* increased significantly at 6 h, reached the peak at 12 h, and then decreased gradually. However, when the peripheral nerve was injured, the target muscle was not directly damaged, and there was no obvious amyotrophic phenotypic change within 3 days, but muscle atrophy was found 7 days after denervation. In order to explain this important functional difference, further flow analysis of gastrocnemius muscle after 3 days of denervation showed that the number of MerTK^hi^Ly6C^lo^ reparative macrophages did not change, regardless of whether *S100a9* was knocked out or not. The same evaluation of gastrocnemius muscle samples found that *S100a9* had no regulatory effect on muscle atrophy within 3 days of denervation. After 3 days of denervation, *S100a9* gradually regulated the complete or partial polarization of MerTK^hi^Ly6C^lo^ reparative macrophages and recruited them to the target muscle to delay the progress of muscle atrophy. It is worth noting that between 3 and 7 days after denervation, MerTK^hi^Ly6C^lo^ reparative macrophages still existed in the target muscle of mice that received *S100a9* KO, although the number is small, indicating that S100A9 is not completely indispensable to the progress of this cell population. In fact, previous study has identified neutrophil gelatinase-associated lipoprotein-troponin (NGAL) as an additional neutrophil secretory factor that can directly stimulate MerTK expression in macrophages^37^. Here, we found that S100A9 promotes the polarization of MerTK^hi^Ly6C^lo^ reparative macrophages by activating transcription factor *Nr4a1*. Therefore, the synergistic effect of S100A9 and NGAL, as well as other undisclosed mediators, is needed to produce reparative macrophages that can most effectively delay the progression of denervated muscle atrophy. We agree that addressing the translational feasibility and challenges of targeting the S100A8/S100A9–macrophage axis is critical for bridging our findings to clinical applications. We summarize the key additions to address these points: therapeutic strategies targeting S100A8/S100A9 inhibition or macrophage reprogramming show translational potential but require careful consideration of feasibility. Small-molecule inhibitors (e.g. paquinimod) and neutralizing antibodies against S100A8/A9, currently in clinical trials for autoimmune disorders such as rheumatoid arthritis, could be pharmacologically repurposed to mitigate muscle atrophy. Parallel approaches to enhancing reparative MerTK^+^ macrophage polarization — through Growth arrest-specific 6 (Gas6) supplementation or Tyro3, Axl, MerTK (TAM) receptor agonists — have demonstrated efficacy in preclinical models of fibrosis and nerve regeneration. However, systemic administration of these agents risks off-target immune modulation, necessitating tissue-specific delivery systems.

The purpose of this study was to explore the functional role and underlying molecular mechanism of neutrophil-macrophage interactions in denervated skeletal muscle atrophy. Our findings revealed that neutrophil-derived S100A8/S100A9 acts as a reparative factor to upregulate Nr4a1 expression. Increased Nr4a1 further facilitates the polarization of MerTKhiLy6Clo reparative macrophages and thereby contributes to attenuating denervated muscle atrophy. It is concluded that a new strategy for delaying denervated muscle atrophy can be formed by targeting S100A8/S100A9 and reparative macrophages. Traditional models of muscle atrophy have focused on myofibre-intrinsic mechanisms or pro-inflammatory immune cells. Our work highlights the dual role of immune cells, where neutrophils drive reparative macrophage polarization via S100A9. This challenges the paradigm of neutrophils as purely detrimental actors and underscores the spatiotemporal complexity of immune interactions in atrophy. Targeting S100A9 signalling (e.g. via small-molecule inhibitors or neutralizing antibodies) could offer a novel strategy to delay muscle atrophy by skewing macrophage responses towards a reparative phenotype. The S100A9/MerTK^+^–macrophage axis may serve as a biomarker for disease progression or therapeutic response in conditions such as spinal cord injury, amyotrophic lateral sclerosis, or critical illness myopathy, where denervation is a key driver of morbidity. Future studies should validate these mechanisms in human tissues and explore combinatorial therapies that simultaneously target neutrophils and macrophages to preserve muscle mass and function. Despite the promising findings presented in this study, the clinical translation of targeting the identified early-stage mechanism for the treatment of amyotrophic lateral sclerosis and spinal cord injury still faces several key practical challenges. Chief among these are the need to develop efficient and precise delivery systems capable of specifically targeting lesion sites during the narrow early pathological window, as well as the urgent requirement for validated and sensitive early biomarkers to accurately stratify eligible patients and guide timely intervention. Future translational investigations will focus on optimizing targeted delivery strategies and conducting clinical correlative analyses to screen reliable early diagnostic markers, which are essential to improving clinical applicability and gradually realizing the therapeutic potential of this target in future clinical practice. The muscle atrophy resulting from sciatic nerve transection does not fully reflect the true clinical and pathological situation. With regard to disuse atrophy (such as that caused by plaster fixation), although it is mainly mediated by mechanical unloading, existing studies have shown that the disuse environment often accompanies a low-grade inflammatory response (low-grade inflammation), and S100A9 is the core mediator of this inflammation. S100A9 is the “key inflammatory mediator of disuse/inflammatory muscle atrophy”^15,38^. Previous studies have clearly demonstrated that S100A9 is a key factor causing ischaemic atrophy (where the nerve is not transected), cancer cachexia (systemic inflammation) and sepsis (severe systemic inflammation) and is a common inflammatory-mediated response observed in various atrophy models (including disuse atrophy)^39–41^. This demonstrates that the increase in S100A9 is not a specific marker of nerve transection but rather a common inflammatory-mediated response observed in various atrophy models (including disuse). Of note, although MerTK^+^ macrophages exert a reliable protective effect and effectively delay the progression of denervated muscle atrophy in the present study, the inherent immunosuppressive properties of MerTK^+^ macrophages inevitably bring potential clinical translational limitations to this intervention strategy. As an important immunomodulatory subgroup, MerTK^+^ macrophages exert prominent anti-inflammatory and immune-suppressive regulatory functions in the body’s microenvironmental homeostasis. Such immunosuppressive characteristics determine that the targeted intervention based on MerTK^+^ macrophages cannot be universally applicable to all patient populations and all disease backgrounds. In particular, the immune-suppressive phenotype of MerTK^+^ macrophages may trigger substantial adverse risks in patients complicated by malignant tumours or other diseases requiring intact and effective immune surveillance and immune clearance function. For patients with tumour comorbidities, the enhanced immunosuppressive effect mediated by MerTK^+^ macrophages may further inhibit the body’s anti-tumour immune response, weaken the immune system’s ability to recognize and clear tumour cells and potentially accelerate tumour progression and metastasis^42,43^. Therefore, the application of MerTK^+^ macrophage-targeted therapy for denervated muscle atrophy requires strict screening of applicable patient groups and rigorous assessment of patients’ underlying disease status. Collectively, these inherent application limitations and potential clinical risks should not be ignored. Fully elaborating these dual aspects of the research intervention helps to present an objective and comprehensive evaluation of the translational value and clinical application prospect of the MerTK^+^ macrophage-based regulatory strategy, and provides a reliable reference for subsequent follow-up optimization of targeted therapeutic regimens and precise clinical application.

In addition, we will clarify some limitations. The reduction of monocyte-derived macrophages observed in our study appears to be context dependent, with preliminary data suggesting a strong association with denervation. However, we acknowledge that specificity requires validation across additional models (e.g. inflammation, tissue injury) to rule out non-specific effects. In our current analysis, denervation-induced neuronal signalling (e.g. loss of neurotrophic factors) probably drives this macrophage subset reduction, but further experiments — such as comparing denervated versus innervated tissues in transgenic models — will be necessary to confirm exclusivity to denervation.

The depletion of haematopoiesis in *S100a9*-deficient mice introduces complexity in interpreting local tissue effects, as systemic haematopoietic defects may indirectly influence macrophage populations in peripheral tissues. To address this, we propose future experiments using conditional KO models (e.g. tissue-specific *S100a9* deletion) to decouple local versus systemic contributions. Additionally, bone-marrow chimeric assays (reconstituting *S100a9*-deficient mice with WT haematopoietic stem cells or vice versa) could dissect whether the observed macrophage reduction is driven by intrinsic haematopoietic defects or local tissue signs.

Although neutrophils are a major source of *S100a9* in bone marrow under basal conditions, emerging literature suggests that other cell types — such as macrophages, dendritic cells and even activated stromal cells — may contribute to *S100a9* expression during inflammation or tissue stress. In our study, we focused on neutrophils owing to their dominant role in acute responses, but we recognize the need to validate cell-specific expression using single-cell RNA sequencing or immunohistochemistry with lineage markers. This will be added as a future direction in the manuscript to acknowledge the complexity of the cellular origins of *S100a9.*

The specific receptors and downstream signalling pathways responsible for S100A9-mediated crosstalk between immune cells and skeletal muscle during reparative muscle remodelling remain to be fully elucidated and these detailed molecular mechanisms will be systematically investigated in our future work.

Future studies employ cast immobilization to directly evaluate the efficacy of S100A9 inhibition in a pure disuse atrophy model.

Although we demonstrate that S100A9 deficiency reduces reparative macrophages and accelerates denervation-induced atrophy, we cannot fully exclude the possibility that S100A9 may also act directly on muscle fibres. The precise signalling pathway — whether S100A9 primarily modulates macrophage polarization, influences muscle-cell function, or both — requires further investigation. Additionally, the unique neuro-muscular microenvironment created by denervation cannot be fully recapitulated in in vitro systems, limiting our ability to dissect direct versus indirect mechanisms. Future studies will focus on clarifying these downstream mechanisms.

## Supplementary information
