# Supplementary material for: S100A8/S100A9 released by CD11b+Ly6G+ neutrophils activate MerTKhiLy6clo reparative macrophages to delay denervated muscle atrophy
Source: Exp Mol Med. 2026 Aug 7;58(8):2733–45. doi: 10.1038/s12276-026-01813-0 (PMC13538589; doi:10.1038/s12276-026-01813-0)
Supplement: Supplementary file 1 [file 12276_2026_1813_MOESM1_ESM.pdf]

Supporting information

Methods

Antibodies and Reagents

Anti-CD45, Ly6G (1A8), CD48 and CD150 antibody were obtained from BioLegend. FVS was obtained from eBioscience. CD115, CD11b, Sca-1, MerTK and Ly6C were obtained from Pharmingen. c-Kit was obtained from Thermo Fisher Scientific. DNase I was obtained from Solarbio. Polyclonal goat anti-mouse Mer anti-body and goat IgG antibody were obtained from R&D Systems.

Cell Preparation, Flow Cytometry and Sorting

Preparation of Gastrocnemius Muscle Single-Cell Suspensions

Gastrocnemius muscle tissue was rinsed with phosphate-buffered saline (PBS), placed in a 6-cm petri dish, and cut into small pieces (2-4 mm). DNase I (1 mg/mL) was mixed with 1640 medium, and 5 ml was added to the tissue pieces. Tissue block solution was added to a gentleMACS C tube, and 5 μl of type II collagenase was added. The tissue block was then placed in the gentleMACS C tube with the enzyme solution, the tube was placed in the casing of the gentleMACS tissue processor, and the heating module was inserted. The 37C-mr-SMDK-1 program was selected for dissociation, and after the program finished running, the C tube was removed and briefly centrifuged to pellet the tissue debris. The resuspended cells were filtered with a 70-μm filter, and the resulting cell suspension was collected in 50-ml test tubes. The filters were rinsed with 10 ml of RPMI 1640 medium, the rinse medium with resuspended cells was centrifuged for 5 min, and the supernatant was discarded. The resuspended cells were then counted and stained.

Preparation of Peripheral Blood Monocyte Suspensions

The mouse was anesthetized with isoflurane. A 1-ml syringe was connected to a No. 7 needle, the syringe was inserted into the inner canthus, and it was rotated toward the fundus. The insertion depth was 2~3 mm. Due to the blood pressure, the blood flows into the tube by itself, and the needle can be pulled out. To prevent puncture hole

bleeding, gauze was used to oppress the eyeball to achieve hemostasis. Blood (0.2 ml) was collected from the mice at one time. A total of 50 μl of anticoagulant was added to the mouse red blood cell lysate, the mixture was diluted to 1 ml (BioLegend, Cat # 420301), it was added to the blood sample, and the sample was incubated at room temperature and away from light for 15 min. The lysate was then centrifuged at 4 °C at 400 × g for 5 min, and the sample was observed for the presence of red blood cells. If red blood cells were observed, 1 ml of red blood cell lysate at room temperature was added while avoiding light and centrifuged at 4 °C for 5 min. Next, 3 ml of PBS was added to the resuspended cells and centrifuged at 4 °C for 5 min. Finally, the cells were resuspended and counted and stained.

Preparation of Spleen Single-Cell Suspensions

The tissue was collected into a buffer containing 10 ml PBS and pressurized with the piston of the syringe to make a single cell suspension. All operations were carried out on ice. The cell screen was placed on top of a 50 ml centrifuge tube. Transfer the cells from the cell petri dish to the centrifuge tube, allowing the cells to flow through the cell sieve and filter out cell lumps and fragments at 4 °C at 400 × g for 5 min, discarded supernatant. Resuspension cells in red blood cell lysate (BioLegend, Cat # 420301) were incubated at room temperature for 8 min and washed twice with 1× PBS. The cells were resuscitated with appropriate volume of PBS solution, and the cell concentration was adjusted to 1×10^6 cells/ml. 1 ml PBS resuspens-ion cells were treated with 1 μl FVS620 staining (BioLegend, Cat # 423109) and incubated at room temperature for 30 min. The supernatant was discarded by centrifugation after adding 3 ml PBS resuscitated cells at 4 °C at 400 × g for 5 min. The resuspended cells were then counted and stained.

Magnetic Bead Sorting

Put the sorting column into the magnetic field of the corresponding MACS separator, and pre-wet the LS separation column with 3 ml buffer. The cells

were labeled with magnetic beads of CD45 and incubated at 4 ℃ without light for 20 min. Add 2 ml buffer, centrifuge wash the cells once, and discard all the supernatants. Cells were counted and the cell concentration was adjusted to 1×10^7 cells/ml. The cell suspension was added to the sorting column to collect unlabeled cell fluid (CD45 negative cells). Wash the sorting column with an appropriate amount of buffer to collect the unlabeled cell sap flow. Remove the sorting column from the sorter and put it on the collection tube. Add 1 ml buffer to the sorting column, push the piston into the sorting column, push it to the bottom at once, and rinse out the magnetic bead labeled cells (CD45 positive cells).

Flow Sorting

The CD45 positive cells were sorted by magnetic beads, the 2ml buffer was resuscitated and precipitated, and centrifuged at 400 × g for 5min. Add 100 µl heavy suspension cell precipitation and 5 µl corresponding antibody dyes respectively to incubate 15 min at room temperature without light. 2 ml buffer was added to resuscitate cell precipitation and 400g was centrifuged for at 400 × g for 5min. The cell concentration was adjusted to 1×10^7 cells/ml. The cell suspension was filtered by 40 µm filter and 10 µl 7AAD was added. The flow machine was adjusted to separate double positive cells and negative cells respectively. The sorted cell suspension was centrifuged at 4 °C at 500 × g for 5 min, then the cells were fully lyzed with 1 ml trizol, frozen in liquid nitrogen and placed in the refrigerator at -80 ℃ for subsequent quantitative real-time PCR analysis.

RNA-Seq

Bioinformatic Analysis

Quality Control and Mapping

Raw data (raw reads) were processed using Trimmomatic (*1*). Reads containing poly-N

and those of low quality were removed to obtain clean reads, which were mapped to the reference genome using hisat2 (*2*).

Identification of Differentially Expressed Genes (DEGs) and Gene Ontology (GO) and Kyoto Encyclopedia of Genes and Genomes (KEGG) Pathway Enrichment Analyses

The fragments per kilobase of transcript per million mapped reads value (*3*) of each gene was calculated using cufflinks (*4*), and the read counts of each gene were obtained with htseq-count (*5*). Differentially expressed genes (DEGs) were identified using the DESeq package of R software with the estimateSize Factors and nbinomTest functions. A P value < 0.05 and fold change > 2 or < 0.5 were set as the thresholds for significantly different expression. Hierarchical cluster analysis of DEGs was performed to explore gene expression patterns. Gene Ontology (GO) enrichment and Kyoto Encyclopedia of Genes and Genomes (KEGG) pathway enrichment analyses of DEGs were performed using R based on the hypergeometric distribution (*6*).

Neutrophil Depletion

Neutrophil depletion was performed via intraperitoneal administration of 200 µg of anti-Ly6G (1A8, Bio X Cell) antibody 24 h prior to injury induction for the SD-IVM experiment**(*7*)**. To evaluate neutrophil function in denervated muscle, C57 BL/6 mice were injected intraperitoneally with an initial 400 µg followed by 100 µg thrice weekly for anti-Ly6G (1A8, Bio X Cell) to deplete neutrophils. Neutrophils were depleted in Ly6g-DTR-GFP mice by intraperitoneal injection of 10 µg/g body weight of diphtheria toxin (DT) thrice weekly(*8*).

References

1. A. M. Bolger, M. Lohse, B. Usadel, Trimmomatic: a flexible trimmer for Illumina sequence data. *Bioinformatics* **30**, 2114-2120 (2014).
2. D. Kim, B. Langmead, S. L. Salzberg, HISAT: a fast spliced aligner with low memory requirements. *Nat Methods* **12**, 357-360 (2015).
3. A. Roberts, C. Trapnell, J. Donaghey, J. L. Rinn, L. Pachter, Improving RNA-Seq expression estimates by correcting for fragment bias. *Genome Biol* **12**, R22 (2011).
4. C. Trapnell *et al.*, Transcript assembly and quantification by RNA-Seq reveals unannotated transcripts and isoform switching during cell differentiation. *Nat Biotechnol* **28**, 511-515 (2010).
5. S. Anders, P. T. Pyl, W. Huber, HTSeq--a Python framework to work with high-throughput sequencing data. *Bioinformatics* **31**, 166-169 (2015).
6. M. Kanehisa *et al.*, KEGG for linking genomes to life and the environment. *Nucleic Acids Res* **36**, D480-484 (2008).
7. J. Wang *et al.*, Visualizing the function and fate of neutrophils in sterile injury and repair. *Science* **358**, 111-116 (2017).
8. S. B. Coffelt *et al.*, IL-17-producing γδ T cells and neutrophils conspire to promote breast cancer metastasis. *Nature* **522**, 345-348 (2015).
